# A Mechanism-Based Approach to Anti-Aggression Psychotherapy in Borderline Personality Disorder: Group Treatment Affects Amygdala Activation and Connectivity

**DOI:** 10.3390/brainsci11121627

**Published:** 2021-12-10

**Authors:** Corinne Neukel, Katja Bertsch, Marc Wenigmann, Karen Spieß, Marlene Krauch, Sylvia Steinmann, Sabine C. Herpertz

**Affiliations:** 1 Department of General Psychiatry, Center for Psychosocial Medicine, Medical Faculty, Heidelberg University, 69115 Heidelberg, Germany; katja.bertsch@psy.lmu.de (K.B.); marc.wenigmann@med.uni-heidelberg.de (M.W.); karen.spiess@gmx.de (K.S.); MarleneElisabeth.Krauch@med.uni-heidelberg.de (M.K.); sabine.herpertz@uni-heidelberg.de (S.C.H.); 2 Department of Psychology, Ludwig-Maximilians-University Munich, 80802 Munich, Germany; 3 Institute of Medical Psychology, Center for Psychosocial Medicine, Medical Faculty, Heidelberg University, 69115 Heidelberg, Germany; 4 Department of Psychosomatic Medicine and Psychotherapy, Central Institute of Mental Health, Medical Faculty Mannheim, Heidelberg University, 68159 Mannheim, Germany; sylvia.steinmann@zi-mannheim.de

**Keywords:** aggressive behavior, borderline personality disorder, psychotherapy, mechanisms of reactive aggression, threat sensitivity, emotional face matching

## Abstract

Aggression is highly prevalent in borderline personality disorder (BPD). Previous studies have identified specific biobehavioral mechanisms underlying aggression in BPD, threat sensitivity being among them. We composited the mechanism-based anti-aggression psychotherapy (MAAP) in order to target these specific mechanisms, and MAAP was found to be superior to non-specific supportive psychotherapy (NSSP) in reducing aggressive behavior. In the present study, we investigated whether underlying brain mechanisms expected to be involved were affected by MAAP. To this end, *n* = 33 patients with BPD and overt aggressive behavior (*n* = 20 in MAAP, *n* = 13 in NSSP) and *n* = 25 healthy participants took part in a functional magnetic resonance imaging emotional face-matching task before and after treatment, or at a similar time interval for controls. Overt aggressive behavior was assessed using the overt aggression scale, modified. Results showed a decrease in amygdala activation in response to facial stimuli after MAAP, whereas an increase in amygdala activation was found after NSSP. Furthermore, in the MAAP group, connectivity between amygdala and dorsomedial prefrontal cortex increased from pre- to post-treatment compared to the NSSP group. Hence, the results suggest an impact of MAAP on brain mechanisms underlying the salience circuit in response to threat cues.

## 1. Introduction

Aggressive behavior, especially reactive aggression, is highly prevalent among female and male patients with borderline personality disorder (BPD) [1]. In a multidimensional model, Mancke et al. [2] identified biobehavioral mechanisms underlying reactive aggression in patients with BPD, including maladaptive anger regulation, deficits in mentalizing, approach rather than avoidance of threat and increased emotional contagion. One fundamental mechanism underlying aggressive behavior is threat sensitivity, processed in the neural threat circuit, with the amygdala playing a central role in processing threat cues [2,3]. Our group developed mechanism-based anti-aggression psychotherapy (MAAP), which aims at reducing aggressive behavior in patients with BPD by specifically targeting the identified biobehavioral mechanisms, including threat sensitivity. In a randomized controlled trial (RCT), MAAP was compared to a non-specific supportive psychotherapy (NSSP) and was found to reduce overt aggressive behavior measured with the primary endpoint overt aggression scale modified (OAS-M, [4]) at 6 months follow-up [5]. Additionally, patients who took part in MAAP showed a clinically relevant decline of 65% in overt aggression from the pre- to post-treatment timepoints, while patients who took part in NSSP showed a decline of 33%. In order to more accurately understand how psychotherapeutic treatments work and to evaluate and optimize treatments, it is particularly important to understand the mechanisms of change, which have to be targeted to arrive at an effective treatment [6]. Therefore, following Kazdin’s recommendations for psychotherapy research [6], we included measures of mechanisms targeted by MAAP in this study in order to be able to evaluate whether they show treatment-related changes. The present work aims at investigating whether a central brain mechanism thought to underlie threat sensitivity is actually affected by MAAP. 

The amygdala plays a central role in the neural threat circuit since it is part of the salience network [7] and is involved in the processing of arousal and threat-related cues [8]. In functional magnetic resonance imaging (fMRI) studies, the neural threat circuit is mostly stimulated by angry and fearful faces. Angry and fearful faces are typically referred to as threat-related faces, as they directly communicate hostility (angry faces) or indirectly hint at a threatening environment (fearful faces). In patients with BPD, enhanced activation (e.g., [9,10], and for a meta-analysis see [11]) of the amygdala in response to highly salient negative facial expressions (e.g., angry, fearful and disgusted faces) has been consistently reported. Additionally, Bilek et al. [12] found reduced habituation of the amygdala in response to angry and fearful facial expressions. Interestingly, in a study combining fMRI and eye-tracking, amygdala reactivity was shown to be related to fast threat responses, i.e., faster saccades to the eyes of angry faces, in a sample of female patients with BPD [13]. Thus, bottom-up threat sensitivity, especially the activation of the salience circuit in response to threat cues that were shown to be of highest salience in patients with BPD, seems to be mainly mediated by the amygdala.

Importantly, amygdala activation also seems to play a role in aggressive behavior. Carré et al. [14] found that in men, the anger trait, which is associated with aggression in BPD [15], is positively correlated with bilateral amygdala reactivity to angry facial expressions. Accordingly, Bertsch et al. [16] reported a correlation between the tendency to act anger out and amygdala activation in male patients with BPD, using an approach–avoidance task. Besides amygdala activation, connectivity of the amygdala with other brain regions was shown to be associated with aggression. Reduced coupling of the amygdala with the middle cingulum was detected in male patients with BPD when imagining narratives of directing physical aggression toward others [17]. Furthermore, previous results showed the reduced connectivity between the amygdala and the ventromedial prefrontal cortex (PFC) to be associated with behavioral reactive aggression in youths with disruptive behavior disorders [18]. This is in line with the finding of reduced coupling of the amygdala with the anterior PFC in male offenders [19]. Together, these results might indicate diminished top-down control of the neural threat circuit in aggressive individuals.

Research investigating the effects of psychotherapeutic treatments on brain mechanisms in patients with BPD is still scarce. First studies indicate that amygdala activation and connectivity can be affected by psychotherapy. Results show that successful dialectical behavior therapy (DBT) is associated with a reduction in amygdala activation as well as an increase in connectivity of the amygdala with the inferior parietal lobe and the fusiform gyrus during reappraisal of negative stimuli [20]. In line with this, patients with BPD showed increased connectivity between the amygdala and the dorsal anterior cingulate in response to negative pictures combined with painful stimuli after DBT, indicating functional emotion regulation processes after DBT [21]. Interestingly, patients with BPD showed reduced symptom severity and less affective instability after completing neurofeedback training in which they were instructed to downregulate amygdala activation [22]. 

In the present study, we aimed at investigating the impact of MAAP on amygdala activation and connectivity. Therefore, we administered an emotional face-matching task during fMRI to assess amygdala activation and coupling with other brain regions in patients with BPD before and after either MAAP or NSSP or in a comparable time interval in healthy participants. First, we expected that MAAP would reduce amygdala activation in response to facial stimuli, especially to angry and fearful faces, from pre-treatment to post-treatment timepoints. Second, we hypothesized that, along with this proposed reduction in amygdala activation, MAAP but not NSSP would increase coupling between the amygdala and other brain regions, especially the PFC. Lastly, we explored whether the change from pre- to post-treatment in amygdala activation and connectivity was associated with the change in overt aggression after MAAP but not after NSSP or in the group of healthy participants.

## 2. Materials and Methods

### 2.1. Trial Design and Study Procedure

The efficacy of MAAP was compared to that of NSSP in a cluster-randomized two-arm parallel-group trial (RCT; for details see [5]). Assessment of overt aggression took place at inclusion and at pre-treatment (T0), post-treatment (T1) and 6 months follow-up (T2) timepoints. Behavioral laboratory (reported elsewhere, e.g., see [23]) and neuroimaging assessments took place at T0 and T1 in order to investigate treatment-related changes in biobehavioral mechanisms. All assessments were conducted by research diagnosticians (clinical assessments) and research assistants (behavioral laboratory and neuroimaging assessments), who were blind to the treatment delivered. 

The study was pre-registered in a public trial archive (German Registry for Clinical Trials, DRKS00009445), performed in accordance with the Declaration of Helsinki and approved by the local ethics committee at the Faculty of Medicine, University of Heidelberg. Participants gave written informed consent before participation and received monetary compensation for taking part in the neuroimaging assessments but not for treatment participation.

### 2.2. Participants

All participants were 18–55 years old. Patients had to meet at least four BPD criteria according to DSM-IV (hence, we also included subthreshold BPD) and had to have an overt aggression and irritability score of at least 6 over a time span of two weeks, according to the OAS-M [4]. Exclusion criteria comprised pregnancy, neurological disorders, current substance abuse (except cannabis) or addiction, additional non-study psychotherapy, bipolar I disorder or schizophrenia, intelligence quotient (IQ) < 70, and change in psychotropic medication within the last three weeks before allocation to trial. Additional exclusion criteria for the fMRI part of the study comprised a positive drug screening prior to an fMRI measurement and medication with benzodiazepines. Originally, 59 patients with BPD were included in the RCT of which 30 were allocated to the MAAP treatment and 29 to the comparator NSSP treatment. Of these patients, 49 completed one of the two treatments and were included in the intent-to-treat (ITT) analysis (for a detailed flow of participants and description of drop outs see [6]). All participants taking part in the RCT were asked to take part in the fMRI part of the study. FMRI data of the emotional face matching task from both the T0- and T1-timepoints were available for 35 of the patients included in the ITT analysis of the RCT [6] since *n* = 4 patients were not willing to take part in the MRI measurement (due to fear), *n* = 6 patients had a positive drug screening prior to one of the MRI measurements, *n* = 3 patients did not take part in the measurement at T1 because they reported the first measurement to be aversive, and *n* = 3 did not have time to take part in the MRI measurement at T1. Furthermore, data were excluded from the analysis due to technical problems (*n* = 1) and change of medication between T0 and T1 (*n* = 1). Thus, the final sample comprised 20 patients with BPD in the MAAP treatment group (MAAP-BPD) and 13 patients with BPD in the NSSP treatment group (NSSP-BPD). Three participants from the MAAP-BPD group completed seven instead of at least eight out of a total of twelve group sessions that were the lower limit to enter per-protocol analyses. Additionally, 25 healthy participants (HP) were included in this study in order to compare amygdala activation and connectivity of the patients with BPD to a group of participants without any current or lifetime psychiatric disorder. Hence, HP were excluded if they currently or lifetime fulfilled criteria for any axis I or axis II disorders or had psychotherapy or psychological counselling. Patients with BPD and HP were matched for age and gender. 

### 2.3. Psychometric and Clinical Assessments

Axis I disorders were assessed at inclusion, using the Structured Clinical Interview for DSM-IV [24] and BPD; antisocial personality disorder and avoidant personality disorder were assessed with the International Personality Disorder Examination (IPDE; [25]). We used items 1–3 (verbal aggression, aggression against objects and aggression against others) of the OAS-M [4] to assess externally directed overt aggression as the primary outcome of the RCT and the Zanarini Rating Scale for Borderline Personality Disorder (ZAN; [26]) to assess the severity of borderline symptoms over the last week. Raven’s Progressive Matrices [27] were administered to estimate IQ.

### 2.4. Treatments

The manualized MAAP and NSSP both comprised 1 individual and 12 group sessions (3–6 patients and 2 therapists) over the course of seven weeks. MAAP specifically addresses biobehavioral mechanisms of reactive aggression in BPD by including elements from DBT and mentalization-based therapy (MBT) and specifically developed app-based exercises. Threat sensitivity was addressed specifically by interventions training discrimination between presence and past, mindfulness training and app-based exercises designed to reduce attentional hypervigilance to threatening social cues. NSSP focuses on the non-specific or “common” factors of psychotherapeutic treatments, including psychoeducational elements and a focus on the therapist’s role of reflective listening, empathy, facilitating affect, therapeutic optimism, and acknowledgement of patients’ resources (see [5] for details of the treatments).

### 2.5. Functional MRI Paradigm

The emotional face-matching paradigm [28] has been shown to robustly elicit amygdala activation across different populations. We used the Duke Neurogenetics Study version of the task [29,30,31] which consists of four task blocks each presenting six face trios, one block each for angry, fearful, surprised, and neutral faces (pictures taken from the Pictures of Facial Affect data bank [32]). Participants are asked to answer via button press whether the left or the right face displayed at the bottom is identical to the face displayed at the top of the screen. Before and between the task blocks, participants are presented with five control blocks, each presenting six trios of simple geometric shapes (circles and ellipses) and participants are asked to answer via button press whether the left or the right shape at the bottom of the screen is identical to the shape at the top. All blocks are preceded by a brief instruction (‘Match Faces’ or ‘Match Shapes’) that lasts 2 s. In the task blocks, each of the six face trios is presented for 4 s with a variable interstimulus interval (ISI) of 2–6 s (total block length: 48 s). In the control blocks, each of the six shape trios is presented for 4 s with a fixed ISI of 2 s (total block length: 36 s).

### 2.6. Functional MRI Data Acquisition

Functional imaging was performed on a 3 T whole-body magnetic resonance scanner (Tim Trio; Siemens) equipped with a 32-channel head coil. In each volume, 40 transverse slices (slice thickness = 2.3 mm) were acquired. A T2*-sensitive gradient echo-planar imaging (EPI) sequence was used (TR 2340 ms, TE 26 ms; flip angle 80°; FOV = 220 × 220 mm; in-plane resolution 2.3 × 2.3 mm). Furthermore, isotropic high-resolution (1 × 1 × 1 mm^3^) structural images were recorded using a T1-weighted sagittally oriented MPRAGE sequence.

## 3. Data Analysis

### 3.1. Demographics, Psychometric Data and Clinical Scores

To compare, demographic and psychometric data between the groups (BPD vs. HP and BPD-MAAP vs. BPD-NSSP) at time of inclusion and at T0 χ^2^- and independent *t*-tests were used.

### 3.2. FMRI Data

FMRI data were preprocessed and analyzed using Statistical Parametric Mapping 12 (SPM 12) software (https://www.fil.ion.ucl.ac.uk/spm/software/spm12/, accessed on 6 March 2020). Preprocessing included realignment and co-registration to the mean functional image. Realigned images were then segmented, spatially normalized to the standard Montreal Neurological Institute (MNI) template and smoothed with an 8 mm full width at half-maximum (FWHM) Gaussian filter. Voxels were resampled during preprocessing to be 2 mm^3^. No dataset had to be discarded from further analysis due to excessive head movements (all scan-by-scan movements ≤ 2 mm in x, y, and z directions).

On the first level, we set up a general linear model (GLM) for each participant with angry, fearful, surprised, and neutral faces and shapes as five separate regressors (convolved with a canonical hemodynamic response function) as well as six regressors for motion parameters to control for slow-drift motions. Each subject’s data set was high-pass filtered (temporal cut-off: 128 s) to remove low-frequency drifts and corrected for serial autocorrelations using first-order auto-regressive functions. Contrasts for facial stimuli (angry, fearful, surprised, and neutral faces) vs. shapes, angry and fearful faces vs. shapes and neutral and surprised faces vs. shapes were calculated for each subject. In a first step, we entered contrast images of facial stimuli (angry, fearful, surprised, and neutral faces) vs. shapes into a one-sample t-test to check for task-related blood oxygenation level dependent (BOLD) activation and into two-sample t-tests to test for differences between the groups at T0. Since we were mainly interested in changes between T0 and T1 to test for treatment effects of MAAP on BOLD activation, we then entered the participant’s individual contrast images into a group (BPD-MAAP, BPD-NSSP, HP) × timepoint (T0, T1) full-factorial model and calculated 2-way group × timepoint interactions, followed by post-hoc tests comparing activation at T0 vs. T1 within the groups. In order to specifically address treatment effects with regard to angry and fearful faces, we likewise entered contrast images of angry and fearful faces vs. shapes into a group x timepoint full-factorial model. We used a region-of-interest (ROI) approach and SPM small volume correction (SVC) of *p*_FWE_ < 0.05 on the respective *p* < 0.01 uncorrected whole-brain statistical maps for our a priori defined ROI (bilateral amygdala) to test our hypothesis. The image of the bilateral amygdala for ROI analyses was anatomically defined according to Neuromorphometrics, Inc. (Somerville, MA, USA) (http://Neuromorphometrics.com/, accessed on 6 March 2020) under academic subscription. Subsequently, a generalized psychophysiological interaction analysis (gPPI; [33]) was performed to explore whether the coupling of the amygdala with other brain regions was affected by MAAP using a full factorial model as described above. We selected the cluster in the amygdala from the contrast identifying changes from T0 to T1 that differed between BPD-MAAP and BPD-NSSP as the seed region for the gPPI analysis. All full-factorial models were also conducted without the outlier on overt aggression (see results for details on the outlier). Parameter estimates (beta weights) were extracted from the first level individual contrasts using the MarsBaR Toolbox [34]. Finally, to evaluate our third hypothesis concerning the association of change in amygdala activation and connectivity with the change in overt aggression, we calculated the difference of the parameter estimates between the pre- and post-treatment timepoints; accordingly, we calculated the difference in the overt aggression scores between pre- and post-treatment. Subsequently, we performed correlational analyses between the calculated difference scores for each group separately, followed by post-hoc power analyses.

## 4. Results

### 4.1. Demographics, Psychometric Data and Clinical Scores

Demographic data, psychometric and clinical characteristics of participants at the time of inclusion and the overt aggression scores at T0 and T1 are shown in Table 1. The patients with BPD randomized to the MAAP treatment did not differ from the patients with BPD randomized to the NSSP treatment with regard to clinical characteristics at time of inclusion. However, they differed with regard to overt aggression at T0, which was mainly due to one patient scoring particularly high at T0 (i.e., the overt aggression score of this patient was more than 1.5 interquartile range from quartile 1). As the OAS-M has no maximum value, this participant was not excluded as an outlier. The MAAP-BPD group showed a decline of 62.2% in overt aggression from pre- to post-treatment, while the NSSP-BPD group showed a decline of 42.3%. At 6 months follow-up, reduction in overt aggression differed significantly between the two treatment groups (*t* (29) = 2.351, *p* = 0.026). Regarding individuals, only one patient in the MAAP-BPD group and six patients in the NSSP-BPD group exhibited a (clinically less relevant) increase in overt aggression at 6 months follow-up. The BPD and the HP groups differed significantly with regard to BPD symptom severity, IQ and overt aggression at time of inclusion (see Table 1).

### 4.2. Task-Related Amygdala Activation at Pre-Treatment Timepoint

In accordance with previous studies using the emotional face-matching task, we found significant activation of the right (*p*_FWE_ < 0.001) and left (*p*_FWE_ = 0.016) amygdala across all participants when presented with facial stimuli compared to shapes. When comparing the BPD group to the HP group, we did not find a significant difference regarding amygdala activity between the groups. Additionally, there was no significant difference in amygdala activation in response to facial stimuli vs. shapes between the MAAP-BPD and the NSSP-BPD groups before taking part in the MAAP or NSSP treatment (see Table 2).

### 4.3. Effect of MAAP on Task-Related Amygdala Activation

**Figure 1:**
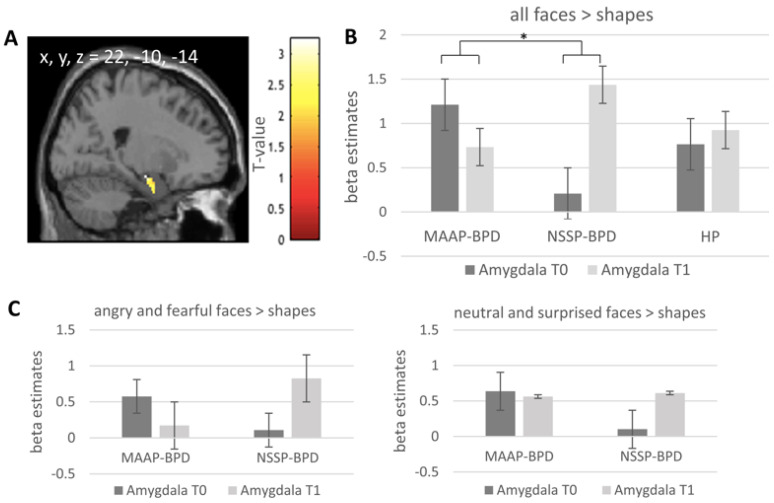
(**A**) Significant cluster (Amygdala; peak voxel x, y, z = 22, −10, −14, k = 11) from the group (MAAP-BPD vs. NSSP-BPD) × Timepoint (T0 vs. T1) interaction for all faces > shapes. The statistical map is overlaid on a single-subject canonical brain image with a display threshold of *p* = 0.05, uncorrected for visualization purposes. (**B**) Group means of beta estimates for all faces > shapes from this cluster from the MAAP-BPD, NSSP-BPD and HP groups are presented, with error bars representing the standard error of the mean. (**C**) Group means of beta estimates for angry and fearful faces > shapes and for neutral and surprised faces > shapes from this cluster from the MAAP-BPD and NSSP-BPD are presented, with error bars representing the standard error of the mean. MAAP—mechanism-based anti-aggression psychotherapy; NSSP—non-specific supportive psychotherapy; BPD—borderline personality disorder; HP—healthy participant; T0—pre-treatment; T1—post-treatment; * *p* < 0.05.

To test for effects of the MAAP treatment, we calculated group (MAAP-BPD vs. NSSP-BPD and MAAP-BPD vs. HP) by time (T0 vs. T1) interactions. In line with our hypothesis, the MAAP-BPD group compared with the NSSP-BPD group showed a significant reduction in right amygdala activation in response to facial stimuli vs. shapes from T0 to T1, while we found the opposite pattern (i.e., an increase of amygdala activation from T0 to T1) in the NSSP-BPD group (*p*_FWE_ = 0.042); see Figure 1A,B. Post hoc-tests of right amygdala activation showed a significant reduction from T0 to T1 in the MAAP-BPD group (*p*_FWE_ = 0.046); the increase in right amygdala activation from T0 to T1 in the NSSP-BPD group did not reach statistical significance (*p*_FWE_ = 0.065). Descriptively, a decrease in this cluster from pre- to post-treatment in the MAAP-BPD group contrasted with an increase in the NSSP-BPD group also in the comparison of angry and fearful faces, while for neutral and surprised faces, an increase can be seen after NSSP but no change in activation in this cluster can be seen after MAAP (see Figure 1C). When comparing amygdala activation in response to angry and fearful faces vs. shapes between the MAAP-BPD and NSSP-BPD groups, the results only revealed a non-significant statistical trend in the left amygdala (*p*_FWE_ = 0.080), indicating a reduction in left amygdala activity from T0 to T1 in the MAAP-BPD group. Post-hoc tests comparing left amygdala activation at T0 and T1 within the groups did not yield significant results. Comparing the MAAP-BPD group with the HP, we found a non-significant statistical trend, too, indicating a reduction in right amygdala activation in response to facial stimuli vs. shapes from T0 to T1 in the MAAP-BPD group (*p*_FWE_ = 0.099; see Table 2 for details), while no significant increase in right amygdala activation was found in a post-hoc test within the HP group. No significant interaction effect comparing the MAAP-BPD and the HP groups was found when only considering the amygdala response to angry and fearful faces vs. shapes in the analysis. The additional analyses without the outlier showed that the findings remained significant when excluding the outlier.

### 4.4. Effect of MAAP on the Coupling of the Amygdala with Other Brain Regions

**Figure 2:**
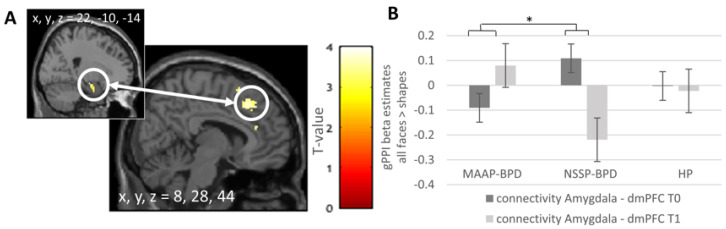
(**A**) Significant cluster (dmPFC; peak voxel x, y, z = 8, 28, 44, k = 224) from the group (MAAP-BPD vs. NSSP-BPD) x Timepoint (T0 vs. T1) interaction of the gPPI analyses (seed: amygdala cluster, peak voxel x, y, z = 22, −10, −14, k = 11) for all faces > shapes. The statistical map is overlaid on a single-subject canonical brain image. (**B**) Group means of gPPI beta estimates from this cluster from the MAAP-BPD, NSSP-BPD and HP groups are presented, with error bars representing the standard error of the mean. dmPFC—dorsomedial prefrontal cortex; gPPI—generalized psychophysiological interaction analysis; MAAP—mechanism-based anti-aggression psychotherapy; NSSP—non-specific supportive psychotherapy; BPD—borderline personality disorder; HP—healthy participant; T0—pre-treatment; T1—post-treatment; * *p* < 0.05.

To test for effects of the MAAP treatment on the coupling between the amygdala and other brain regions, we calculated group by time interactions as described above (see Figure 2). Confirming our hypothesis, we found an increase in coupling between the right amygdala and dorsomedial PFC (dmPFC—cluster extending to supplementary motor area) for facial stimuli vs. shapes from T0 to T1 in the MAAP-BPD group compared to the NSSP-BPD group (*p*_FWE_ = 0.010). Additionally, compared to HP, we found increased coupling between the amygdala and the cerebellum in the MAAP-BPD group at T1 versus T0 (*p*_FWE_ = 0.008). No significant changes from T0 to T1 concerning the coupling of the amygdala and other brain regions were found when analyzing only the neural response to angry and fearful faces vs. shapes; see Table 3. The additional analyses without the outlier showed that findings remained significant when excluding the outlier.

### 4.5. Correlation of Change in Amygdala Activation and Connectivity with Change in Overt Aggression

Analyses did not show significant correlations between changes in brain mechanisms and changes in overt aggression from pre- to post-treatment, neither in the MAAP-BPD, the NSSP-BPD nor the HP groups (see Table 4). Post-hoc power analyses revealed a power of 0.16 for the correlation between change in amygdala activity and overt aggression and a power of 0.18 for the correlation between change in connectivity of the amygdala with the dmPFC in the MAAP-BPD group.

## 5. Discussion

This is the first study that investigated the impact of a mechanism-based approach to anti-aggression psychotherapy compared to the impact of non-specific supportive psychotherapy on the central brain mechanism thought to underlie threat sensitivity. The results indicate a significant reduction in amygdala activation in response to facial stimuli from pre- to post-treatment timepoints in patients who took part in MAAP compared to those who took part in NSSP. Additionally, we found increased coupling between the amygdala and the dmPFC in the MAAP-BPD compared to the NSSP-BPD group and between the amygdala and the cerebellum in the MAAP-BPD compared to the HP group. No significant correlations were found between pre- to post-treatment change in amygdala activation and connectivity and change in overt aggression. However, the reduction in overt aggression differed significantly between the MAAP-BPD and the NSSP-BPD group at 6 months follow-up.

The finding of a reduction in amygdala activation from pre- to post-treatment in response to facial stimuli in patients with BPD who took part in MAAP is in accordance with our a priori hypothesis. We found a significant effect when considering all facial stimuli and a non-significant trend when considering angry and fearful faces only. The non-significant trend when considering only angry and fearful faces in the analysis might be due to less power when including two instead of all four (angry, fearful, surprised and neutral faces) task blocks in the analysis since, descriptively, a decrease in activation in this cluster in the MAAP-BPD group can be seen also for angry and fearful faces only but not for neutral and surprised faces only, while an increase in activation can be described for all faces in the NSSP-BPD group (see Figure 1C). Thus, the activation pattern hints at a specific effect for highly salient angry and fearful faces in the MAAP-BPD group while a non-specific effect for all faces can be seen in the NSSP-BPD group. This non-specific effect of increased activation in response to all faces in the NSSP-BPD group is likely to contribute to the group x timepoint interaction effect when considering all faces vs. shapes becoming significant, while the power to detect a significant interaction effect is not sufficient when only two task blocks (i.e., angry and fearful faces) instead of all four (angry, fearful, surprised and neutral faces) are included in the analysis. In addition to the reduction in amygdala activation after MAAP and confirming our second hypothesis, we also found an increased connectivity between the amygdala and the dmPFC in response to facial stimuli from pre- to post-treatment in the MAAP-BPD group. The dmPFC is implicated in the attribution of mental states [35], and increased coupling of the amygdala and the dmPFC has consistently been demonstrated to play a role in the downregulation of emotions (for meta-analysis see [36]). In line with this, individuals who trained to downregulate amygdala activation using neurofeedback showed increased connectivity of the amygdala with the dmPFC after four training sessions [37]. Hence, the present result of an increased connectivity at T1 in patients who participated in MAAP might indicate facilitated top-down control of amygdala activation in response to facial stimuli and, hence, a possible top-down modulation of the salience circuit activated by threat cues. Additionally, we found increased coupling from T0 to T1 between the amygdala and the cerebellum in the MAAP-BPD group compared to the HPs. Functional connectivity between the amygdala and the cerebellum is well reported [38,39] and the cerebellum was thus discussed to be engaged in the modulation of emotional processing [40]. Interestingly, functional as well as structural abnormalities of the cerebellum were previously identified in patients with BPD and might, thus, play a role in disturbed emotion processing in BPD [11], while speculatively increased connectivity between the amygdala and cerebellum after MAAP might indicate improved functional processing of facial stimuli. No significant treatment-related changes concerning the coupling of the amygdala and other brain regions were found when analyzing only the neural response to angry and fearful faces vs. shapes. Again, this is likely to be due to reduced power when including two instead of all four task blocks in the analysis.

The current findings indicate that MAAP can affect underlying neural mechanisms and thus provide deeper insight into the change process, although results have to be interpreted with caution due to the small sample size for which we cannot confirm an association between change in neural mechanisms and change in aggressive behavior. Several interventions of MAAP may have contributed to affect amygdala activation and connectivity: The app-based attentional tasks were designed to reduce attentional hypervigilance to threatening social cues, which, together with mindfulness training, might be reflected in reduced amygdala activation in response to facial stimuli. Additionally, MAAP affects cognitive processes, as participants train to regulate irritability and anger via DBT techniques and to mentalize others’ mental states. Improved top-down control interacting with threat sensitivity might be represented by increased connectivity between amygdala and dmPFC. Importantly, the present results are in line with a previous study that found a reduction in amygdala activation and increased connectivity between the amygdala and the inferior parietal lobe (a region associated with emotion regulation [41]) in patients with BPD who successfully participated in DBT [20]. In the present study, the lack of significant correlations poses the question of whether the reduction in overt aggression is indeed accompanied by a change in amygdala activation and connectivity. It is possible that this is due to non-sufficient power (post-hoc power analyses showing only a small power for correlations in the MAAP-BPD group), the more so as we found on the behavioral level, that change in response latencies to facial expressions correlated with change in overt aggression [23]. Therefore, results should be confirmed in a bigger sample.

Interestingly, compared to the MAAP-BPD group, patients with BPD who participated in NSSP showed an opposite pattern, i.e., increased amygdala activation and reduced connectivity between the amygdala and the dmPFC in response to facial stimuli after treatment. This might be due to the therapist’s role facilitating affect and, thus, NSSP rather increasing sensitivity to emotional stimuli on the neural level, reflected in increased amygdala activation and reduced connectivity of the amygdala with the dmPFC. Importantly, the MAAP-BPD and NSSP-BPD groups did not differ with regard to amygdala activation before treatment. In contrast to the two treatment groups, HP participants (who did not participate in a therapeutic treatment) did not show an increase or decrease in amygdala activation in response to facial stimuli at T1 compared to T0. Regarding amygdala activation in response to facial stimuli at the pre-treatment timepoint, it has to be considered that in the present study, in contrast to previous reports of neural activation in response to facial stimuli, we did not find a significant difference between patients with BPD and HP. Since the emotional face matching task robustly elicits strong amygdala activation in various samples, including samples with HP [42], this might be due to a ceiling effect. Furthermore, a meta-analysis by Schulze et al. [11] reported that only currently medication-free samples of patients with BPD showed amygdala hyperactivity, which does not apply to the sample included in our study.

In summary, in this sub-sample of the RCT included in the fMRI analysis, we found a significant reduction in overt aggression at follow-up and a change in brain mechanisms underlying and interacting with the salience circuit activated by threat cues at post-treatment timepoint in the MAAP-BPD group, but, contrary to our expectations, we did not find an association of neural change with change in overt aggression. Since the reduction in overt aggression corresponds to the results of the ITT analysis in the larger sample [5], we would expect the findings from the fMRI analyses to show even stronger effects in a larger sample. In order to detect correlations between the change in overt aggression with the change in brain mechanisms, a larger sample might be needed, too. Furthermore, despite the results supporting our a priori hypotheses regarding the impact of MAAP on a central brain mechanism underlying threat sensitivity, some limitations of the study have to be mentioned. First, due to the complex study design requiring fMRI measurements before and after a 7-week group psychotherapy program, the group sizes were relatively small, so results should be interpreted with caution and replicated in a larger sample. Second, the group of patients with BPD was heterogenous regarding psychotropic medication, with some patients being medicated and others not. We cannot rule out that the medication status of the patients may have influenced neural activation in response to facial stimuli. However, medicated patients were randomized to both treatment groups, and medication with benzodiazepines was among the exclusion criteria. Importantly, by not excluding medicated patients with BPD, the generalizability of the study findings to real-world settings is enhanced. Third, three patients included in the present analyses completed 7 instead of at least 8 out of a total of 12 group sessions that were the lower limit to enter per-protocol analyses. Lastly, despite the randomization procedure, the overt aggression scores of the MAAP-BPD and the NSSP-BPD group differed at the pre-treatment timepoint. Thus, we cannot exclude the possibility that MAAP impacts amygdala activation and connectivity, especially in patients with high scores of overt aggression at treatment start. However, at time of inclusion, no difference in overt aggression between the two groups was found, and a stable treatment effect (reduction of overt aggression) at 6 months follow-up in the MAAP-BPD, but not in the NSSP-BPD group, was reported in the RCT [5], too, underlining the superiority of MAAP over NSSP. FMRI measurements were not performed at the follow-up timepoint, but in psychotherapy, change in mechanisms is assumed to precede change in symptoms [6,43].

In conclusion, by showing an impact of MAAP on amygdala activation and connectivity, the present study highlights that brain mechanisms underlying and interacting with the salience circuit in response to threat cues are affected by anti-aggression group psychotherapy in patients with BPD. Bottom-up as well as top-down processes seem to be influenced by MAAP and may impact aggressive behaviors in everyday life, underlining their relevance as therapeutic targets. Since knowledge on mechanisms of change is highly important for understanding how psychotherapy works, the present results may be used to further optimize anti-aggression therapy for patients with BPD.

## Figures and Tables

**Table 1:** Demographic and psychometric information of patients with BPD (randomized into MAAP or NSSP treatment) and HP.

	BPD				Group Comparison			HP	Group Comparison		
					(MAAP vs. NSSP)				(BPD vs. HP)		
	MAAP-BPD M (SD)/N (%)		NSSP-BPD M (SD)/N (%)		t (df)/χ	*p*	Cohen d/φ	M (SD)/N (%)	t (df)/χ	*p*	Cohen d/φ
Age	30.2 (10.0)		30.5 (10.2)		−0.073 (31)	0.943	−0.026	30.2 (7.8)	0.043 (56)	0.966	0.011
Gender (female)	15 (75)		9 (69.2)		0.132	0.716	0.063	18 (72)	0.004	0.951	0.008
IQ	102.7 (3.4)		99.7 (5.5)		0.488 (30)	0.629	0.177	115.0 (10.4)	−3.513 (54)	0.001	−0.949
ZAN (Total)	14.1 (5.0)		12.7 (2.9)		0.900 (30)	0.375	0.325	0.1 (0.4)	17.363 (31.9)	<0.001	4.130
overt aggression at inclusion	44.0 (35.3)		30.5 (20.3)		1.244 (31)	0.223	0.444	0.2 (0.4)	7.229 (32.0)	<0.001	1.664
overt aggression at T0	41.8 (40.0)		16.3 (18.3)		2.479 (28.5)	0.019	0.765	-	-	-	
overt aggression at T1	15.8 (20.6)		9.4 (7.1)		1.073 (30)	0.292	0.383	0.9 (1.7)	4.131 (31.9)	<0.001	0.960
overt aggression at T2	12.5 (14.9)		27 (47)		−0.993 (11.1)	0.342	−0.461	1.2 (2.1)	3.007 (30.4)	0.005	0.720
Current psychotropic medication											
Antidepressants	6 (30)		4 (30.8)		0.002	0.961	−0.009	-			
Neuroleptics	3 (15)		3 (23.1)		0.269	0.604	0.092	-			
Other	2 (10)		1 (7.7)		0.073	0.787	−0.048	-			
Comorbidities	current	lifetime	current	lifetime							
Major depression	5 (25)	15 (75)	3 (23.1)	11 (84.6)	-	-		-			
Dysthymia	3 (15)	-	2 (15.4)		-	-		-			
Alcohol addiction/abuse	0	3 (15)	0	4 (30.8)	-	-		-			
Anxiety disorders	11 (55)	8 (40)	4 (30.8)	6 (46.2)	-	-		-			
Obsessive-compulsive disorder	1 (5)	1 (5)	0	0	-	-		-			
Post-traumatic stress disorder	7 (35)	6 (30)	4 (30.8)	4 (30.8)	-	-		-			
Somatization Disorder	1 (5)	-	0	-	-	-		-			
Eating Disorders	3 (15)	3 (15)	1 (7.7)	1 (7.7)	-	-		-			
Antisocial Personality Disorder	3 (15)	5 (25)	0	1 (7.7)	-	-		-			
Avoidant Personality Disorder	3 (15)	3 (15)	4 (30.8)	4 (30.8)	-	-		-			

BPD—borderline personality disorder; HP—healthy control; MAAP—mechanism-based anti-aggression psychotherapy; NSSP—non-specific supportive psychotherapy; T0—pre-treatment; T1—post-treatment; T2—6 months follow-up; IQ—intelligence quotient; ZAN (total)—total score of the Zanarini Rating Scale for Borderline Personality Disorder.

**Table 2:** Task-related amygdala activation at pre-treatment timepoint, group (MAAP-BPD vs. NSSP-BPD and MAAP-BPD vs. HPs) by timepoint (T0 vs. T1) interactions and post-hoc tests within the groups.

Timepoint	Contrast	Cluster Size (k)	T Value	*p*_FWE_ Value	Peak Voxel MNI: x, y, z (mm)
all faces > shapes					
Pre-treatment	all	77	4.86	<0.001	24, 0, −22
		51	3.68	0.026	−24, −6, 16
	BPD > HP	8	2.8	0.133	24, −6, −24
	MAAP-BPD > NSSP-BPD	11	2.98	0.118	−26, −10, −16
T0 vs. T1	(MAAP-BPD > NSSP-BPD) × (T0 > T1)	11	3.24	0.042	22, −10, −14
		27	2.99	0.078	−30, −8, −24
	MAAP-BPD: T0 > T1	4	2.94	0.046	24, 0, −22
	NSSP-BPD: T1 > T0	8	2.80	0.065	26, −8, −16
	(MAAP-BPD > HP) × (T0 > T1)	12	2.89	0.099	24, 0, −22
	HP: T1 > T0	-	-	-	-
angry and fearful faces > shapes					
Pre-treatment	all	53	4.24	0.003	20, −6, −14
		50	3.81	0.012	−26, −6, −16
	BPD > HP	-	-	-	-
	MAAP-BPD > NSSP-BPD	-	-	-	-
T0 vs. T1	(MAAP-BPD > NSSP-BPD) × (T0 > T1)	29	3.01	0.080	−26, −6, −15
	MAAP-BPD: T0 > T1	-	-	-	-
	NSSP-BPD: T1 > T0	-	-	-	-
	(MAAP-BPD > HP) × (T0 > T1)	9	2.66	0.175	−22, −4, −16

Peak *p*_FWE_ values after applying SPM small volume correction. MAAP—mechanism-based anti-aggression psychotherapy; NSSP—non-specific supportive psychotherapy; BPD—borderline personality disorder; HP—healthy control; T0—pre-treatment; T1—post-treatment.

**Table 3:** gPPI results for group (MAAP-BPD vs. NSSP-BPD and MAAP-BPD vs. HPs) by timepoint (T0 vs. T1) interactions with seed amygdala (peak voxel: x, y, z = 22, −10, −14; k = 11).

Contrast	Cluster Size (k)	T Value	*p*_FWE_ Value	Peak Voxel MNI: x, y, z (mm)	Anatomical Location of Peak Voxel
all faces > shapes					
(MAAP-BPD > NSSP-BPD) × (T1 > T0)	224	3.96	0.010	8, 28, 44	dorsomedial prefrontal cortex
(MAAP-BPD > HP) × (T1 > T0)	236	4.47	0.008	8, −46, −40	Cerebellum
angry and fearful faces > shapes					
(MAAP-BPD > HP) × (T1 > T0)	-	-	-	-	-
(MAAP-BPD > NSSP-BPD) × (T1 > T0)	-	-	-	-	-

MAAP—mechanism-based anti-aggression psychotherapy; NSSP—non-specific supportive psychotherapy; BPD—borderline personality disorder; HP—healthy control; T0—pre-treatment; T1—post-treatment.

**Table 4:** Correlation of change in brain mechanisms and change in overt aggression from T0 to T1.

	Treatment Change in Overt Aggression					
	MAAP-BPD		MAAP-NSSP		HP	
	r, p	N	r, p	N	r, p	N
**Treatment change in amygdala activity**	−0.238, 0.341	18	−0.019, 0.951	13	0.033, 0.881	23
**Treatment change in connectivity amygdala-dmPFC**	0.261, 0.295	18	−0.511, 0.074	13	0.017, 0.939	23

MAAP—mechanism-based anti-aggression psychotherapy; NSSP—non-specific supportive psychotherapy; BPD—borderline personality disorder; HP—healthy control.

## Data Availability

The data presented in this study are available on request from the corresponding author.
